# Shear Stress Regulates TRPV4 Channel Clustering and Translocation from Adherens Junctions to the Basal Membrane

**DOI:** 10.1038/s41598-017-16276-7

**Published:** 2017-11-21

**Authors:** Sara Baratchi, Markus Knoerzer, Khashayar Khoshmanesh, Arnan Mitchell, Peter McIntyre

**Affiliations:** 1 School of Health and Biomedical Sciences, RMIT University, Melbourne, VIC 3083 Australia; 2 School of Engineering, RMIT University, Melbourne, VIC 3001 Australia

## Abstract

Localized Ca^2+^ influx via TRPV4 on the surface of endothelial cells greatly influences endothelial adaptation to blood flow, but how mechanical stress from blood flow controls TRPV4 integration into this physiological function is not fully understood. Here, we studied the spatial organization of TRPV4 and its relationship to the adherens junction component β-catenin using single- and dual-color direct stochastic optical reconstruction microscopy (dSTORM). In non-stimulated endothelial cells, TRPV4 is clustered in small protein islands, as is β-catenin. Using dual-color imaging, we found that TRPV4 and β-catenin reside in similar islands and can be found at both the basolateral and basal membranes. Following shear stress stimulation, TRPV4 molecules formed smaller clusters, with the majority residing outside of clusters. Further shear stress stimulation changed the molecular distribution of TRPV4 molecules, limiting them to the basal membrane. This redistribution and the smaller clusters resulted in the segregation of TRPV4 from β-catenin. Furthermore, TRPV4 trafficking was controlled by focal adhesion kinase and activation of the α5ß1 integrin. These highly differentiated spatial redistributions suggest that mechanotransduction of blood flow is controlled via a more complex hierarchy than previously thought.

## Introduction

Fluid shear stress associated with blood flow plays a pivotal role in vascular remodelling, arterial and venous identity and angiogenesis^1^. In endothelium, mechanotransduction acts through conversion of step-like physical forces into biochemical information in a series of rapid switch-like events that control many aspects of development and physiology^2^. Cell adhesion molecules are strongly implicated in the mechanotransduction of blood flow^3^. Previous studies suggested that mechanotransduction of blood flow is transmitted through the cell adhesion proteins at adherens junctions to the basal membrane, which stimulate the association and dissociation of mechanosensitive integrins and extracellular matrix (ECM), indirectly and through signaling pathways. New binding between the integrins and ECM is proposed to be essential for long-distance, downstream signaling events^4^.

In the vascular endothelium, localized Ca^2+^ influx through mechanosensitive cation channels plays an important role in endothelial adaptation to flow dynamics^2^. The Transient Receptor Potential (TRP) family of ion channels is the major class of Ca^2+^ permeable ion channels in the endothelium^5^. An increase in [Ca^2+^]_i_ level following TRP channel gating leads to various effects on vascular function such as change in vascular tone, alteration in vascular permeability, change in blood coagulation, oxidative damage and vascular remodelling^6^. Within seconds of shear stress stimulation, Ca^2+^ influx into the cytoplasm through shear stress-dependent Ca^2+^ channels, such as TRPV4, activates inward-rectifying Ca^2+^-sensitive K^+^ channels that co-activate with the outward-rectifying Cl^−^ channels^7^. These events repolarize the membrane, eventually leading to hyperpolarization, which is transmitted through myoendothelial gap junctions to the adjacent smooth muscle cells^8,9^. Furthermore, opening of TRPV4 in endothelial cells and intact endothelium results in localized Ca^2+^ sparklets^10,11^. These sparklets generate subcellular microdomains rich in Ca^2+^, which can activate a variety of Ca^2+^-dependent signaling cascades^11^.

We have previously shown that in HEK293 cell stably expressing TRPV4 (TRPV4-HEK293) shear stress activates TRPV4 and leads to increase in [Ca^2+^]_i_ level in a does dependent manner^12–14^. Further in bovine aortic endothelial cells and human umbilical cord endothelial cells (HUVECs), we have shown that shear stress sensitizes the response of TRPV4 to its selective agonist^12,15,16^ and in HUVECs, shear stress increases the exocytosis of functional TRPV4 channels to the cell membrane^16^.

On the cell membrane, TRPV4 interacts with ß-catenin at adherens junctions, linking them to the actin cytoskeleton^17^. In keratinocytes, TRPV4 expression is essential for the normal cell-cell junctions of skin epithelium^17^. An increase in [Ca^2+^]_i_ disrupts the adherens junction via activation of myosin light-chain kinase and the RhoA-Rho kinase pathway and induces actin stress fiber formation^18,19^. Here, we studied relative molecular distribution and interaction of TRPV4 channels with ß-catenin after shear stress stimulation, using single- and dual-color direct stochastic optical reconstruction microscopy (dSTORM) in HUVECs.

We found that TRPV4 channels are expressed in preclustered structures, composed of 20–25 molecules per cluster, and in a complex with β-catenin. After exposure to shear stress, we have observed relocation of TRPV4 channels. Upon shear stress stimulation, TRPV4 channels formed smaller clusters, with the majority of them relocated from the basolateral membrane to basal membrane, and TRPV4 lost its interaction with β-catenin. The shear-induced translocation of TRPV4 channels was controlled by focal adhesion kinase and α4ß1/α5ß1 integrin.

## Results

### TRPV4 channels exist in nanoclusters and shear stress stimulation increases the density of channels that are not in the cluster

To determine the molecular distribution of TRPV4 channels at the cell membrane, we used a super-resolution fluorescence microscopy technique, dSTORM. We labeled TRPV4 channels expressed in HUVECs with anti-TRPV4 antibodies directly conjugated to a photoactivatable fluorescent dye, Alexa 647.

We analyzed Alexa 647-labeled TRPV4 channels stimulated with 10 dyn/cm^2^ of shear stress and compared them with TRPV4 channels expressed in the absence of shear stress (Control).

**Figure 1:**
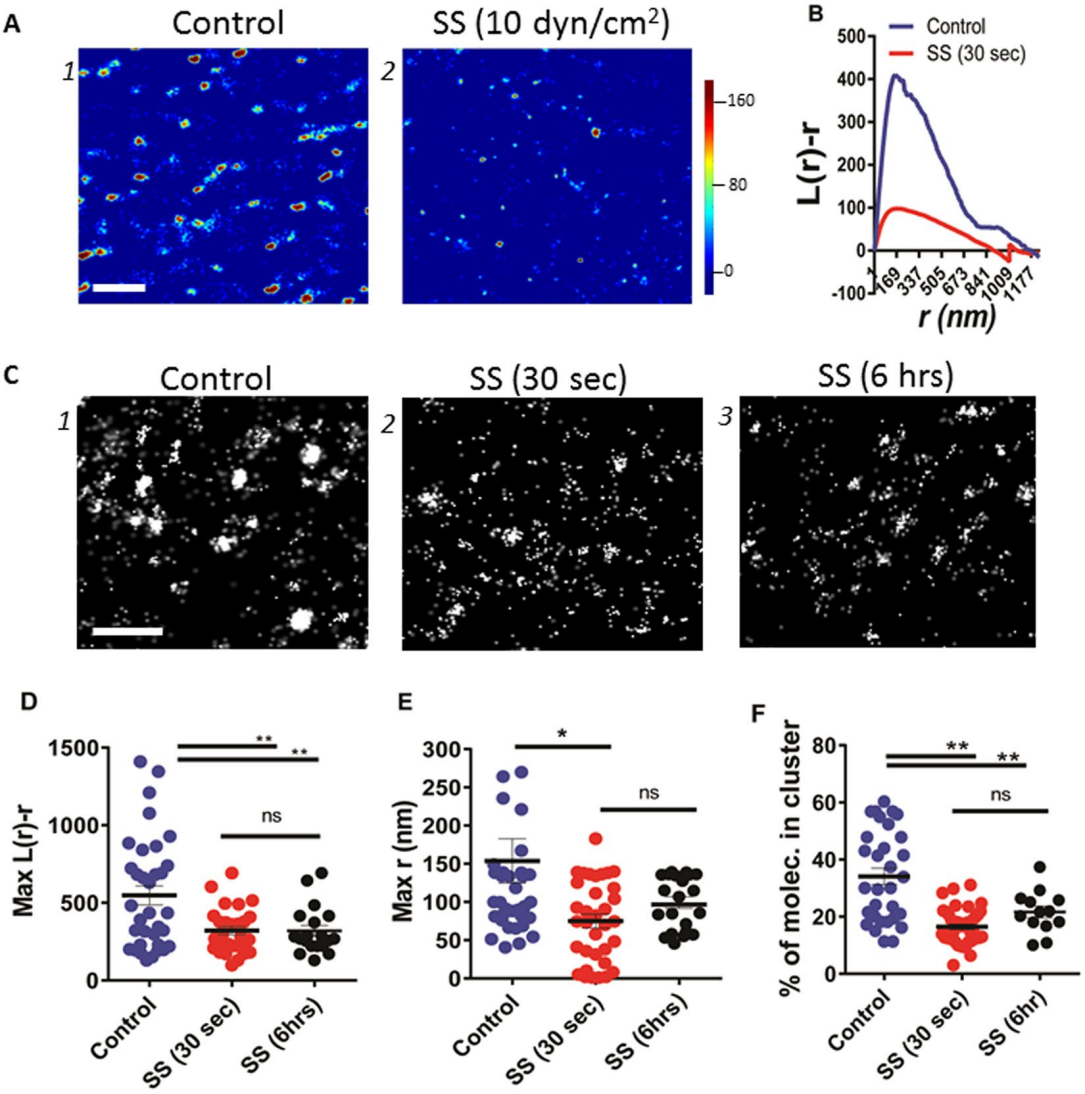
Analysis of the spatial distribution of TRPV4 molecules in HUVECs by dSTORM. (**A**) Color map probability density plots for TRPV4 molecules labelled with anti-TRPV4 antibody directly conjugated to Alexa 647 when resting (**A1**) or after shear stress stimulation (10 dyn/cm^2^ for 30 sec) (**A2**). Scale bar is 1 µm. (**B**) Ripley’s K-function plot of TRPV4 molecules in the resting state (1) or after shear stress stimulations (2). (**C**) dSTORM images of TRPV4 molecules labeled with Alexa 647-anti-TRPV4 at resting and after shear stress stimulations. Scale bar is 500 nm. Peaks of Ripley’s K-function plots were analyzed for the maximum values of L(*r*)-*r* (**D**), *r*_max_ (**E**) and % of cells in cluster (**F**) for multiple cells. (n = 34 and 27 for resting and shear stress activated cells, respectively). *P < 0.01 and **P < 0.0001 (Student’s t-test). The data are representative (**A** and **B**) and collections (**D**–**F**) of experiments for the selected areas of 5.6 × 5.6 µm^2^.

We reconstituted the super resolution images as probability density plots of the tagged molecules (Fig. 1A) and used Ripley’s K function for quantitative spatial analysis (Fig. 1B–D). As reported for other ion channels^18,20,21^, TRPV4 channels were not randomly distributed on the plasma membrane but clustered in nanometer-scale membrane compartments (Fig. 1B).

We examined the effects of both transient and sustained shear stress on molecular distribution of TRPV4 on the membrane. For this, we stimulated endothelial cells grown inside the microfluidic channel for 30 sec or 6 hrs. By comparing the maxima of L(*r*)−*r* in Ripley’s *K*-function analysis (Control *vs* SS), we observed that the degree of clustering decreased by 226.7 ± 73.83-fold (P < 0.01) and 228.5 ± 86.9-fold (P < 0.01) upon 10 dyn/cm^2^ SS stimulation for 30 sec and 6 hrs, respectively (Fig. 1D) and noted that the majority of molecules reside outside of clusters. The *r*_max_ value where L(*r*)−*r* has a maximum value was indicative of the size of the analyzed clusters. The *r*_max_ values also decreased from 153.7 ± 28.8 nm for the control to 73.6 ± 9.3 nm after 10 dyn/cm^2^ SS stimulation for 30 sec (Fig. 1E). Thus, we could conclude that there was a significant decrease in clustering of TRPV4 channels after 10 dyn/cm^2^ SS stimulation compared with the control condition.

The percentage of TRPV4 molecules within a cluster decreased from 34.12 ± 2.87 for the control to 16.41 ± 0.92 and 21.63 ± 2.05 following SS stimulation for 30 sec and 6 hrs, respectively (Fig. 1F).

The decrease in the number of molecules for similar clustering distances before and after SS stimulation further supported the decrease in clustering of TRPV4 molecules upon SS stimulation. We found that stimulation of cells with 1 dyn/cm^2^ SS did not affect the L(*r*)−*r and r*_max_ values, suggesting that SS responses of TRPV4 are stimulus-dependent (Supplementary 1).

### Dual-color STORM reveals that SS stimulation relocates TRPV4 channels from adherens junctions and reduces the interaction of TRPV4 with ß catenin

**Figure 2:**
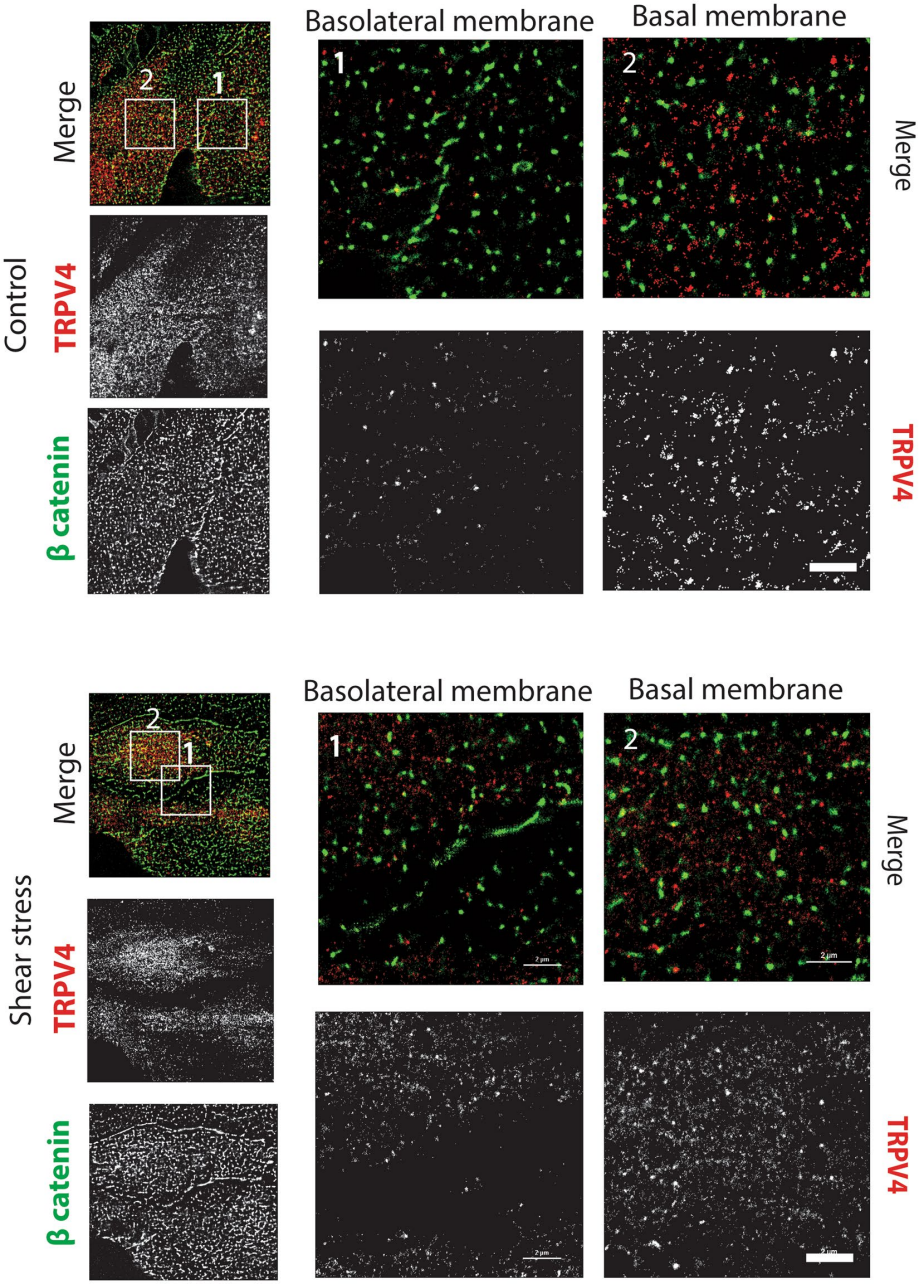
Shear stress stimulation changed the membrane distribution of TRPV4 channels. Reconstructed dual-color dSTORM images of HUVECs before (**A**) and after (**B**) shear stress stimulation. Cells were labeled with anti-TRPV4 antibody directly labeled with Alexa 647 and anti-β catenin antibody directly labeled with Alexa 488. (**A**) In the absence of shear stress, TRPV4 could be found at the (**A1**) basolateral membrane and the (**A2**) basal membrane. After shear stress stimulation (**B**), TRPV4 relocated from the (**B1**) basolateral membrane and accumulated at the (**B2**) basal membrane. Scale is 2 um.

To assess the colocalization of TRPV4 with ß catenin (a component of adherens junctions) in the endothelium, we used dual-color STORM. HUVECs were fixed and permeabilized, and TRPV4 and ß catenin were subsequently labeled with the fluorophore-conjugated monoclonal antibodies anti-TRPV4-Alexa 647 and anti-ß catenin-Alexa 488. Before switching most of the dyes to the dark state for dSTORM acquisition, we used diffraction-limited total internal reflection fluorescence microscopy (TIRFM) and acquired an image of TRPV4. In TIRFM images, TRPV4 were homogeneously distributed in the absence of SS, residing in small nanoclusters. ß catenin also appeared homogeneously distributed, with significant fluctuation of fluorescence signals observed around the basolateral membrane. After shear stress stimulation, the majority of TRPV4 relocated to the basal membrane and was absent from the cell borders, while ß catenin molecules were still homogeneously distributed (Fig. 2A.1). This localization suggests that interaction of TRPV4 with ß catenin following shear stress stimulation was limited to the basal membrane.

TRPV4 formed a complex with ß catenin in adherens junctions of keratinocytes. Given that TRPV4 is a Ca^2+^-permeable ion channel and that increase in [Ca^2+^]_i_ disrupts adherens junctions, we hypothesized that TRPV4 may play a regulatory function in modulating endothelial membrane permeability function. Therefore, we examined the interaction of TRPV4 with ß catenin in endothelial cells by TIRFM and dSTORM after specific fluorescence labeling with polyclonal anti-ß catenin antibody.

First, in TIRFM imaging, we observed some clustered structures of ß catenin molecules for both Control and SS stimulation cases. In non-activated cells, the majority of TRPV4 and ß catenin molecules resided together in nanoclusters that were composed of 11.6 and 12.6 molecules of TRPV4 and ß catenin, respectively. The interaction between TRPV4 and ß catenin was at both the basolateral and basal membrane sites (Fig. 2A 1,2). In SS-stimulated endothelial cells, TRPV4 nanoclusters were absent from the adherens junction sites, and the concatenation of these nanoclusters occurred without molecular interaction between TRPV4 and ß catenin, which enhanced segregation of these molecules from each other (Fig. 2B 1,2).

**Figure 3:**
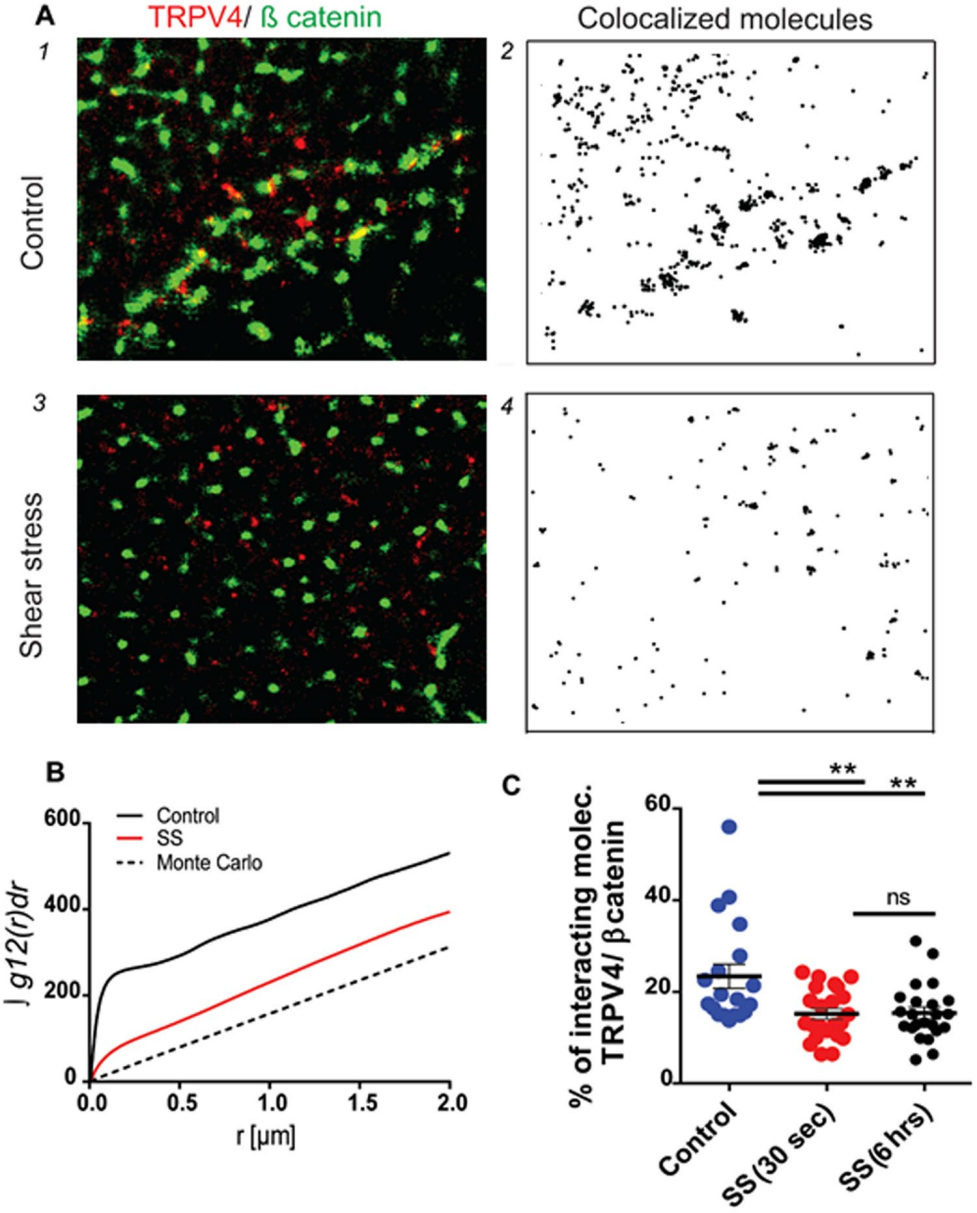
Analysis of the relative spatial distribution of TRPV4 and ß catenin in HUVECs by dual-color dSTORM. (**A**) Dual-color dSTORM images for TRPV4 (red) and ß catenin (green) labeled with Alexa 647-anti-TRPV4 and Alexa 488-anti-ß catenin. The black dots (**A 2** and **4**) represent the probability density distribution of colocalized or interacting molecules for both fluorescent channels. (**B**) Integrals of bivariate pair-correlation function curves of TRPV4 and ß catenin in resting (black line) and shear stress-stimulated (red line) conditions. Black dashed lines indicate 95% confidence intervals of a random labeling (100 Monte Carlo simulations). (**C**) The degrees of interaction between TRPV4 and ß catenin, comparing resting and shear stress-stimulated cells (n = 34 and 27 for resting and shear stress-stimulated cells, respectively). **P < 0.001 (Student’s *t*-test). The data in A and B are representative of 5.6 × 5.6 µm^2^ areas, and the minimum density of 357 molecules per square micrometer was used for the determination of the degree of colocalization (**C**). Scale bar is 2 um.

Furthermore, we confirmed these findings by quantitative analysis. We used bivariate cross-correlation function analysis and calculated the fraction of TRPV4 molecules that had at least one neighboring ß catenin molecule (Fig. 3B). In the absence of shear stress, the curve was above the confidence interval, suggesting that there was a strong interaction between TRPV4 and ß catenin molecules. After shear stress stimulation, the interaction between the two molecules decreased. To quantify the difference in the amount of interaction between TRPV4 and ß catenin after stimulation with shear stress, we compared the density of TRPV4 molecules that were residing within the maximum distance of 5 pixels with ß catenin as a degree of interaction. Using this approach, we found that shear stress stimulation reduced the interaction of TRPV4 and ß catenin molecules by 8.2 ± 2.7 fold (P < 0.001) after 30 sec (Fig. 3C).

Further, we examined the effect of sustained shear stress on spatial redistribution of TRPV4 and its interaction with ß-catenin by stimulating endothelial cells grown inside the microfluidic channel for 6 hrs. Using dual color dSTORM we found that similar to transient stress (30 sec SS stimulation), the sustained stress (6 hrs SS stimulation) also significantly reduces the interaction of TRPV4 with ß-catenin (Fig. 3C).

### Mechanotransduction through β1 integrins controls the trafficking of TRPV4 channels to the cell membrane

**Figure 4:**
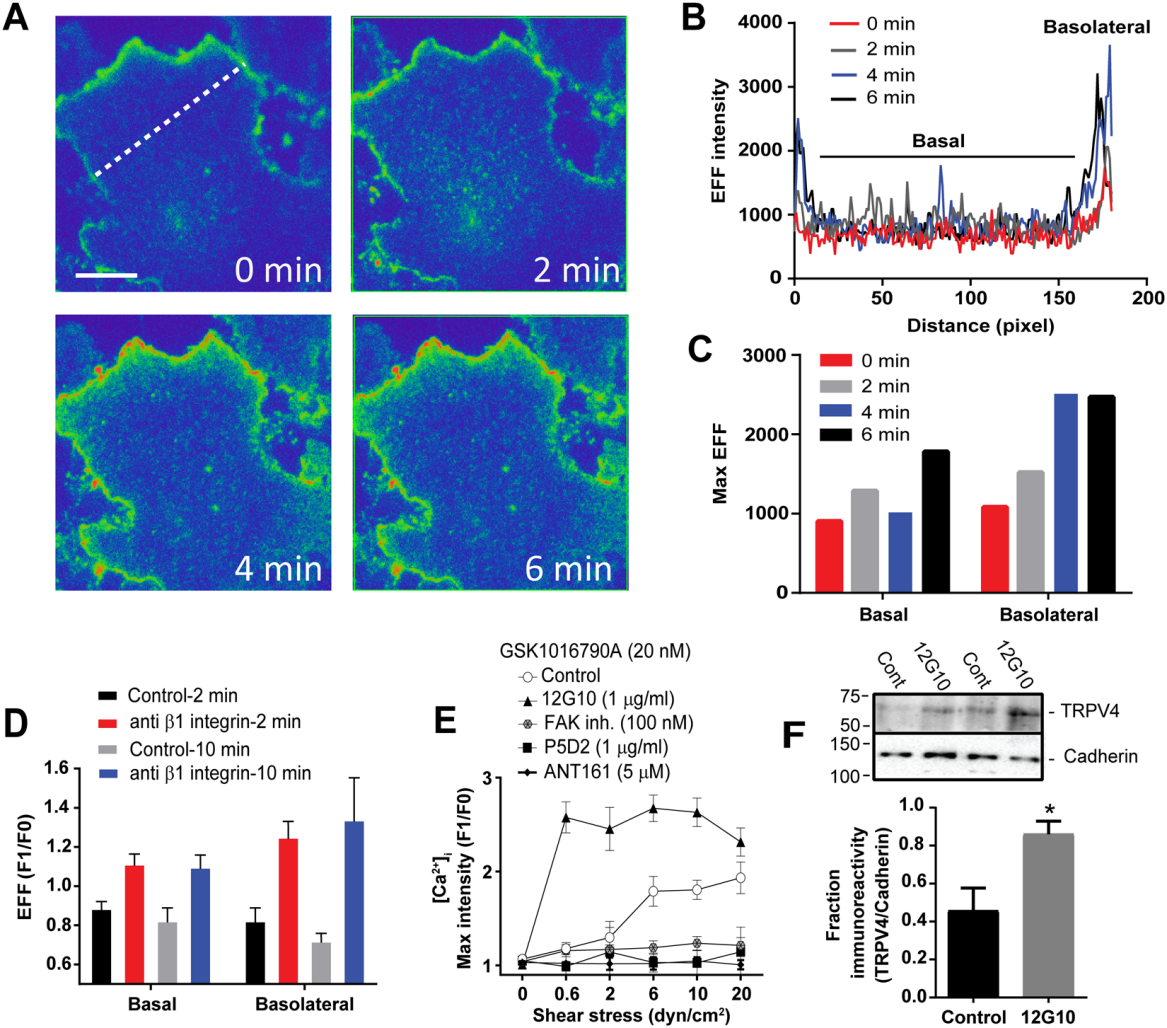
Activation of α5ß1 integrins with activating antibody stimulated the recruitment of TRPV4 channels to the plasma membrane. (**A**) Representative TIRFM images recorded from single HEK293 cells stably expressing Venus-TRPV4 (scale bar is 10 µm). (**B**) Time course of changes in fluorescent intensity across the dashed line in (**A**). (**C**) Time course of changes in max fluorescent intensity of the cell shown in A at both the basal and the basolateral membranes. (**D**) Average changes in maximal fluorescence intensity after 2 and 10 min of stimulation with α5ß1-activating antibody at both the basal and the basolateral membranes. (**E**) Agonist sensitization assay showing that inhibition of focal adhesion kinase and ß1 integrins with focal adhesion kinase inhibitor (PF573228), anti-ß1 integrin antibody (P5D2) and a non-RGD-based integrin binding peptide (ATN-161), respectively, blocked the sensitization effect of shear stress on the TRPV4 response to its selective agonist, while activation of α5ß1 integrin with anti-ß1 integrin antibody (12G10) stimulated the effect of shear stress. (**F**) Cell surface biotinylation assay showing that activation of α5ß1 integrin with anti-ß1 integrin antibody (12G10) increases the cell surface expression of TRPV4 in HUVECs (supplementary 2).

Several reports implicate a role for integrins in mechanotransduction of stimulus from blood flow^2^. Mechanotransduction through β1 integrins controls the trafficking of different membrane proteins to different subcellular compartments^22,23^. In the endothelium, αvβ3 and α5β1 play important roles in the mechanotransduction of blood flow^24^. Previously, we showed that shear stress stimulation controls the rapid trafficking of TRPV4 channels to the cell membrane and showed that trafficking was independent of αvβ3^16^. Here, to examine the hypothesis that mechanotransduction through α4β1 or α5β1 integrin controls the cell surface expression of TRPV4, we used HEK293 cells stably expressing TRPV4 channels fused to Venus protein. Venus-TRPV4 fluorescence was measured in real-time at the sarcolemma by TIRFM. First, in TIRFM images, we observed clusters of TRPV4 channels at both the basolateral and the basal membranes. Upon stimulation, using an anti-α5β1 integrin-activating antibody (clone 12G10), we found progressive increases of the evanescent field intensity at both the basal and the basolateral membranes (Fig. 4A–C). The relative evanescent field fluorescence at the start of the experiment was 0.8 ± 0.1, and it increased to a plateau of 1.2 ± 0.1 (n = 10) 4 min post-addition of the antibody (Fig. 4C).

To investigate the insertion of functional TRPV4 channels, we used Ca^2+^ imaging and a sensitization assay, as previously reported^16^. Using this approach, we found that an increase of the shear stress level from 0.6 to 20 dyn/cm^2^ sensitized the response of TRPV4 to its selective agonist GSK1016790A (10 nM) by 0.8 ± 0.1-fold (P < 0.001, n = 5) at 20 dyn/cm^2^. We found that pretreatment of endothelial cells with 100 nM of FAK inhibitor blocked the sensitization effect of shear stress on the TRPV4 response to GSK1016790A (P < 0.001, n = 5). Furthermore, activation of α5β1 using the 12G10 antibody to β1 integrin stimulated the effect of shear stress on TRPV4 responses to its agonist by 0.83 ± 0.18-fold (P < 0.01, n = 6) at 10 dyn/cm^2^. Inhibition of β1 integrins using P5D2 antibody or ATN-161, a non-RGD- based peptide, mimetic of fibronectin^25^, reduced the sensitization effect of shear stress to the basal level (0.56 ± 0.13-fold (P < 0.01, n = 6) or 0.79 ± 0.13-fold (P < 0.01, n = 5) respectively) (Fig. 4E). The anti-β1 integrin antibody (clone 12G10) stimulates the binding of ligands to the α4β1 and α5β1 integrins by stabilizing their active conformational states^26^. The anti-β1 integrin antibody (clone 12G10) inhibits the binding of β1 integrin to fibronectin^27^.

Further, we used cell surface biotinylation assay to investigate the effect of anti-α5β1 on membrane expression of TRPV4 in HUVECs. Using this approach, we found that stimulation of HUVECs with anti-α5β1, clone 12G10, increases the membrane expression of TRPV4 by 0.38 ± 0.14 (p < 0.05, n = 6) fold, compared to unstimulated endothelial cells (Fig. 4F).

## Discussion

Here, we examined the molecular distribution of TRPV4 on the membrane using super-resolution microscopy. First, we investigated the distribution of endogenous TRPV4 in human endothelial cells using antibody staining. In the absence of shear stress, TRPV4 channels resided in nanoscale clusters or protein islands, as previously shown for Nav and Kv channels at the nodes of Ranvier in neurons^20^, Kv2.1 channels in neurons and transfected human embryonic kidney cells^21^, and MscL channels in lipid bilayers^18^.

Upon shear stress stimulation, these TRPV4 clusters dispersed into individual channels. Since this event coincided with Ca^2+^ influx via TRPV4 into the cytoplasm^16^, we hypothesize that TRPV4 channel activity might be modulated by protein interactions within the clusters.

The mechanotrnsduction, mechanisms by which cells sense and respond to mechanical stress involves the conformational changes of protein domains that are subject to such stress.

β -cathenin is the main adapter protein of the adherens junctions at the point of cell-cell contact that is proposed to transmit the shear stress to the cell interior and cytoskeleton. In particular, it is proposed that tension regulates the protein domains of the β-cathenin that is involved in cell-cell junctions or its cytosolic domain in order to stabilize the cellular adhesion site and regulate the downstream signaling event^28^.

Structurally, β-cathenin is modular protein that contains 12 characteristic armadillo repeats in its central region, R1 to R12, from N- to C-termini that mediates protein-protein interaction. The armadillo repeat domain contains three tyrosine phosphorylation sites that could modulate β-cathenin binding to α-actinin and cadherins. β-cathenin is very sensitive to small changes in mechanical stress, unlike other components of adherens junctions, such as cadherins^28^. Thus, β-cathenin is proposed to act as mechanical buffer which can preserve a link between the cadherin ectodomain and the actin cytoskeleton by absorbing energy through unfolding. Such conformational changes might affect the interaction of β-cathenin with other signaling proteins and receptors. In keratinocytes, TRPV4 interacts with β catenin via its N-terminus to armadillo repeat domain of β-catenin^17^.

Because TRPV4 molecules bind to β catenin at keratinocytes, we expected that in endothelial cells, they might also form such a complex. Here, using dSTORM imaging, we found that only 23% of TRPV4 molecules were in a complex with β catenin, while the rest were segregated. This finding was quantitatively supported by cross-correlation function analysis and by analysis of the degree of interaction, which showed that the determined probability of co-clustering was higher than for randomized events.

To analyze TRPV4-β catenin pairs in shear stress-stimulated endothelial cells, we treated endothelial cells with 10 dyn/cm^2^ of shear stress in a transient manner for 30 sec or in a sustained manner for 6 hrs. Previously we showed that this intensity and duration of signalling was sufficient to stimulate the cell surface expression of functional TRPV4 channels^24^. Shear stress stimulation resulted in segregation of TRPV4 channels from β catenin counterparts and led to the relocation of the channels to the basal membrane. We anticipated that this relocation might be accompanied by formation of new complexes. However, upon analysis of further interactions with other membrane proteins, such as eNOS, Cav-1 and β1-integrins, we could not detect any significant complex formation with those molecules before or after shear stress stimulation.

In endothelial cells, the transmembrane immunoglobulin family protein, PECAM-1, in a mechanosensory complex with VE-cadherin and transmembrane tyrosine kinase vascular endothelial growth factor (VEGF) receptor, Flk-1, mediates the endothelial responses to flow^2^. PECAM-1 mediates homophilic adhesion, and through its cytoplasmic domain, it binds to the β and γ-catenins. Shear stress induces the association of β-catenin and PECAM-1 with the PI(3)K p85 subunit that is dependent on the adaptor protein, VE-cadherin^2^. The formation of this signaling complex correlates with PI3K and AKT activation and regulates β1-integrin activity in endothelium^29,30^.

Here, the observed relocation of TRPV4 after shear stress stimulation suggests that TRPV4 may have two different functions. First, via interaction with β1-integrins, it may act as a scaffolding protein that controls the localization of β catenin in adherens junctions. Second, TRPV4 relocation and activity may control RhoA activity and cytoskeletal remodeling. Adherens junctions in the endothelium play important roles in maintaining endothelial membrane permeability function and in regulating the mechanotransduction of blood flow^18^. RhoA activity is stimulated by Ca^2+^ influx into the cytoplasm^19^_,_ thereby facilitating cytoskeleton remodeling and formation of adherens junctions. The relocation of TRPV4 from adherens junctions to the basal membrane and the increase in [Ca^2+^]_i_ via TRPV4 and RhoA activation supports the concept that shear-induced relocation of TRPV4 from the adherens junction to the basal membrane, and localized Ca^2+^ influx via TRPV4, causes the prolonged activity of Rho family signaling molecules that accelerate cytoskeleton remodeling and control endothelial membrane permeability.

In summary, TRPV4 and β catenin were preclustered in the same protein islands or nanoclusters. Stimulation with shear stress changed the distribution of TRPV4 and confined them to the basal membrane, where channels lost their interaction with the adherens junction component, β catenin. Changes in TRPV4-β catenin localization post-shear stress stimulation suggest that TRPV4 might negatively regulate the membrane permeability of endothelial cells. Furthermore, the relocation of TRPV4 was regulated via β1 integrin in an outside-in signaling pathway.

## Methodology

### Antibody labelling

Anti-TRPV4 (LS-Bio) and anti-ß catenin (Abcam) antibodies were labeled with Alexa 647 and Alexa 488 (Invitrogen), respectively, according to the supplier’s instructions. The labeling efficiencies of TRPV4 and ß catenin were determined to be 0.7–1 dye/protein by absorption spectroscopy assay.

### Cells and reagents

Human umbilical vein endothelial cells (HUVECs) were cultured in complete endothelial growth medium-2 (EGM-2) according to the supplier’s instructions (Lonza). Cells were incubated at 37 °C in a humidified incubator gassed with 95% air and 5% CO_2_, passaged every 3–4 days, and used for up to 5 passages.

HEK293 Flipin T-Rex cell lines (Life Sciences) stably expressing Venus-TRPV4 were generated as reported elsewhere^15,16^ and cultured in Dulbecco’s modified Eagle’s medium supplemented with 10% (v/v) fetal bovine serum (FBS), hygromycin (50 µg/ml), and blasticidin (5 µg/ml).

For fixed-cell experiments, cells were rinsed in phosphate-buffered saline (PBS), fixed for 10 min in 4% paraformaldehyde (PFA) at room temperature, and subsequently washed three times in PBS before imaging.

### Western blotting and cell surface biotinylation assay

Western blotting and cell surface biotinylation assay as reported before^16^.

### Total internal reflection fluorescence microscopy (TIRFM)

All TIRFM experiments were performed on a Nikon Ti microscope controlled by Nikon Elements software. The microscope was fitted with a 100**× **TIRF objective; a 1.49 numerical aperture; an Andor iXon electron multiplying charge coupled device (EMCCD) camera; 405-, 491-, 561- and 633-nm laser lines; an appropriate quadrant multiband laser filter (Semrock); a programmable mechanized stage; and infrared autofocus (Perfect Focus).

HEK293 cells stably expressing Venus-TRPV4 were plated on chamber slides (Ibidi µ-Slide VI 0.4) and induced with 0.1 µg/ml tetracycline for 12 hours. Before each experiment, the growth medium was replaced with imaging buffer (Hank’s balanced salt solution (HBSS) containing: 140 mM NaCl, 5 mM KCl, 20 mM HEPES, 11 mM D-glucose, 1 mM MgCl_2_, 2 mM CaCl_2_, 2 mM probenecid and 10% FBS, pH 7.4.). Live-cell TIRFM imaging experiments were performed and analyzed as reported in detail elsewhere^16^.

### Super-resolution microscopy (dSTORM)

dSTORM imaging was performed on a Nikon N-STORM system based on a Nikon Eclipse Ti microscope with a 100× /1.46 N.A lens (Nikon, Japan). TRPV4, ß catenin or ß1 integrin samples stained with Alexa 647-TRPV4, Alexa 488 ß catenin or ß1 integrin were excited by a 640-nm and 488-nm lasers using a Quadrant bandpass filter. Photo-conversion rates were accelerated using a 405-nm UV diode laser (Radius405, Coherent) in epifluorescence mode. Fluorescence emissions from Alexa 647 and Alexa 488 were separated by a quadrant multiband laser filter (Semrock). The stream acquisitions of images were obtained using a back-illuminated EMCCD camera (Cascade II:512, Roper Scientific) at a readout speed of 5 MHz with full electron multiplying (EM) gain, allowing continuous photo conversion and excitation of the samples.

For dSTORM imaging, the endothelial cells in culture medium were loaded into the flow chamber (Ibidi) and incubated in a CO_2_ tissue culture incubator overnight. Subsequently, cells were stimulated with a shear stress of 10 dyn/cm^2^ and were fixed with ice-cold 4% (vol/vol) formaldehyde and 0.2% (vol/vol) glutaraldehyde in PBS for 30 min, washed and blocked with 2% (vol/vol) bovine serum albumin (BSA) in PBS, and permeabilized with 0.2% (vol/vol) Triton X-100 in PBS for 30 sec. After incubation with antibodies for labeling and rigorous washes, the chamber was exchanged with an oxygen scavenging buffer (50 mM Tris, 10 mM NaCl, 200 mM β-mercaptoethylamine, 10% (wt/vol) glucose, 0.56 mg/mL glucose oxidase, 40 μg/mL catalase) for dSTORM imaging. The images were acquired at a frame rate of 20–50 ms for approximately 5,000–20,000 frames. Drift correction and single-molecule fitting were performed with the N-STORM software in NIS Elements (version 4.13.01).

### Data analysis

dSTORM data was analyzed using custom software written in MATLAB (MathWorks) for a region of 5.6 × 5.6 µm^2^; events with localization precision worse than 50 nm were discarded. All acquisition frames were corrected for any drift, including mechanical, thermal, chromatic aberrations and frameshifts, using the drift correction function available in NIS Elements software.

Strict thresholds were applied for signal-to-noise ratio, intensity, full width at half maximum, and the positional accuracy to filter the signals determined to represent single molecules. Furthermore, to avoid duplicate detection of the same molecule, all the signals located within the same pixel in consecutive frames were discarded except for the first frame of the particular series. The reconstructions of dSTORM images were performed using NIS Elements software.

Before computing the heat map, the data were capped for maximum and minimum values so that scaled color bars could be displayed. The heat map was generated with MATLAB’s hist3 function and interpolated with the MATLAB function imresize to smoothen the contours. The contour map was a heat map with two values. Ripley’s K and L functions were calculated using a function provided by the University of Colorado http://www.colorado.edu/geography/class_homepages/geog_4023_s07/labs/lab10/RipleysK.m].

The bivariate pair-correlation function was performed with a function provided by the MathWorks community [https://au.mathworks.com/matlabcentral/fileexchange/31353-two-point-correlation-function-of-a-finite-2d-lattice]. As a null hypothesis, we used a random labeling model in which all the molecules were randomly labeled for a given number of molecules similar to the test groups and the locations of random events were preserved^31^. The positive deviation of the data line from the lower confidence line indicated a statistically co-complexion behavior compared with random labeling.

### Shear stress experiments, sensitization assay and data analysis

Shear stress experiments were performed as reported previously^16^.

## Electronic supplementary material

Supplemantary material
