# Supplementary material for: Shear Stress Regulates TRPV4 Channel Clustering and Translocation from Adherens Junctions to the Basal Membrane
Source: Sci Rep. 2017 Nov 21;7:15942. doi: 10.1038/s41598-017-16276-7 (PMC5698423; doi:10.1038/s41598-017-16276-7)
Supplement: Supplementary file 1 — Supplemantary material [file 41598_2017_16276_MOESM1_ESM.pdf]

Shear Stress Regulates TRPV4 Channel Clustering and Translocation from Adherens Junctions to the Basal Membrane

Sara Baratchi[1], Markus Knoerzer[2], Khashayar Khoshmanesh[2], Arnan Mitchell[2], Peter McIntyre[1]

[1] School of School of Health and Biomedical Sciences, RMIT University, VIC 3083, Australia

[2] School of Engineering, RMIT University, VIC 3001, Australia

Corresponding author:
Dr. Sara Baratchi, School of Health and Biomedical Sciences, RMIT University, Melbourne, Australia

Email: sara.baratchi@rmit.edu.au

Supplementary 1

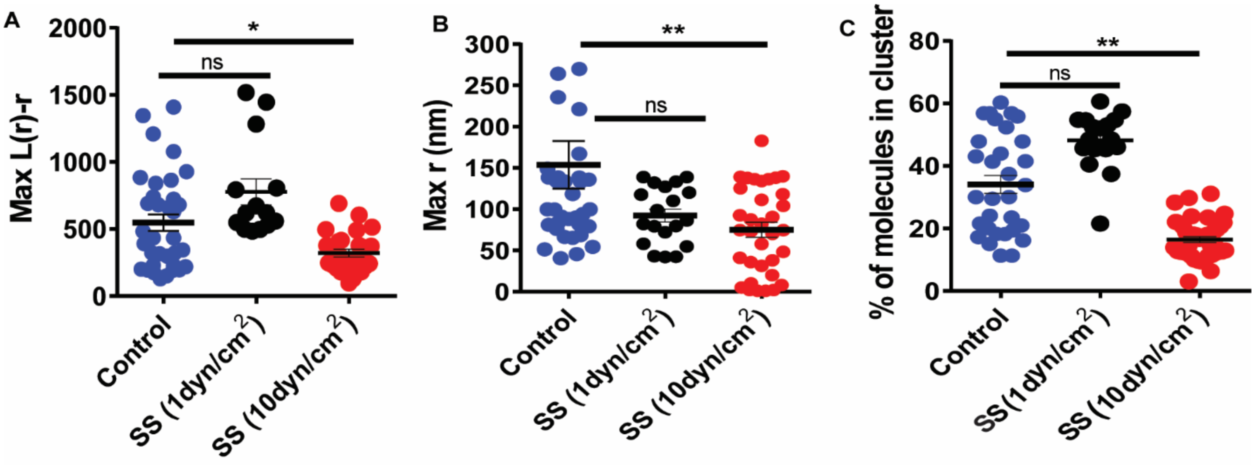

Supplementary 1 Stimulation of HUVECs with a low shear stress of 1 dyn/cm^2 did not change the spatial distribution of TRPV4. Peaks of Ripley's K-function plots were analyzed for the maximum values of L(*r*)-*r* (A), r_{max} (B) and % of molecules in cluster (C) for multiple cells.

Supplementary 2

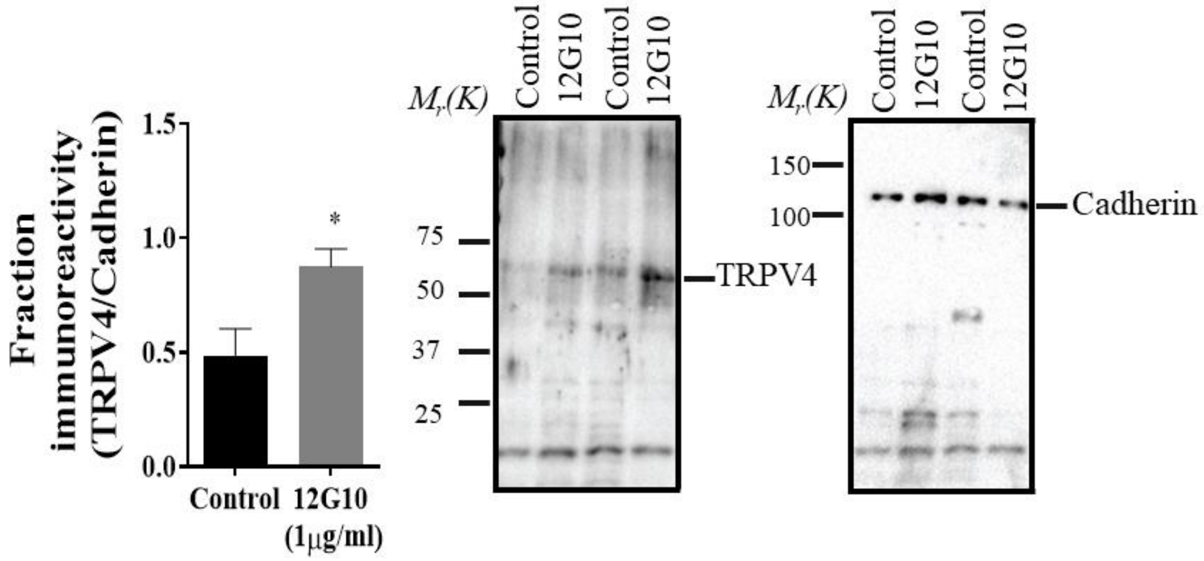

Supplementary 2 Cell surface biotinylation assay showing that activation of α5ß1 integrin with anti-ß1 integrin antibody (12G10) increases the cell surface expression of TRPV4 in HUVECs.
